# A Better Anti-Diabetic Recombinant Human Fibroblast Growth Factor 21 (rhFGF21) Modified with Polyethylene Glycol

**DOI:** 10.1371/journal.pone.0020669

**Published:** 2011-06-06

**Authors:** Zhifeng Huang, Huiyan Wang, Meifei Lu, Chuanchuan Sun, Xiaoping Wu, Yi Tan, Chaohui Ye, Guanghui Zhu, Xiaojie Wang, Lu Cai, Xiaokun Li

**Affiliations:** 1 School of Pharmacy and Chinese-American Research Institute for Diabetic Complications, Wenzhou Medical College, Wenzhou, Zhejiang, China; 2 Normal Bethune Medical College, Jilin University, Changchun, Jilin, China; 3 Key Laboratory of Ministry of Education for Tissue Transplantation and Immunology, Jinan University, Guangzhou, Guangdong, China; 4 Department of Pediatrics, University of Louisville, Louisville, Kentucky, United States of America; University of Hong Kong, China

## Abstract

As one of fibroblast growth factor (FGF) family members, FGF21 has been extensively investigated for its potential as a drug candidate to combat metabolic diseases. In the present study, recombinant human FGF21 (rhFGF21) was modified with polyethylene glycol (PEGylation) in order to increase its *in vivo* biostabilities and therapeutic potency. At N-terminal residue rhFGF21 was site-selectively PEGylated with mPEG20 kDa-butyraldehyde. The PEGylated rhFGF21 was purified to near homogeneity by Q Sepharose anion-exchange chromatography. The general structural and biochemical features as well as anti-diabetic effects of PEGylated rhFGF21 in a type 2 diabetic rat model were evaluated. By N-terminal sequencing and MALDI-TOF mass spectrometry, we confirmed that PEG molecule was conjugated only to the N-terminus of rhFGF21. The mono-PEGylated rhFGF21 retained the secondary structure, consistent with the native rhFGF21, but its biostabilities, including the resistance to physiological temperature and trypsinization, were significantly enhanced. The *in vivo* immunogenicity of PEGylated rhFGF21 was significantly decreased, and *in vivo* half-life time was significantly elongated. Compared to the native form, the PEGylated rhFGF21 had a similar capacity of stimulating glucose uptake in 3T3-L1 cells *in vitro,* but afforded a significantly long effect on reducing blood glucose and triglyceride levels in the type 2 diabetic animals. These results suggest that the PEGylated rhFGF21 is a better and more effective anti-diabetic drug candidate than the native rhFGF21 currently available. Therefore, the PEGylated rhFGF21 may be potentially applied in clinics to improve the metabolic syndrome for type 2 diabetic patients.

## Introduction

Fibroblast growth factors (FGF) are widely expressed in the fetal and adult tissues [1] and play crucial roles in multiple physiological functions, including angiogenesis, mitogenesis, pattern formation, cell differentiation, metabolic regulation and tissue injury repair [2]. The FGF family consists of 22 members [3], among which FGF19, FGF21, and FGF23 belong to one subfamily that exerts regulation of bile acid, cholesterol, glucose, vitamin D and phosphate homeostasis in a klotho-dependent endocrine manner [4]–[6].

FGF21 is expressed in the liver and thymus [7], adipose tissue [8] and pancreatic islet β-cells [9]. Expression of FGF21 can be up-regulated in skeletal muscle in response to Akt activation [10]. The role of FGF21 in metabolic regulation was first discovered in association with its adipocyte-specific capacity of causing glucose uptake, which is accomplished in part by up-regulating transcription of the glucose transporter GLUT1. Administration of recombinant human FGF21 (rhFGF21) lowered plasma glucose and insulin levels, reduced hepatic and circulating triglycerides and cholesterol levels, and improved insulin sensitivity, energy expenditure, and obesity in a variety of insulin resistant animal models [5], [11], [12]. Furthermore, FGF21 administration led to a significant improvement of lipoprotein profiles, by lowering low-density cholesterol and raising high-density cholesterol, and losing body weight in the animals [11]. Hence, rhFGF21 has become an attractive candidate potentially to treat human type 2 diabetes and associated metabolic syndrome. Several laboratories have produced rhFGF21, but its expression levels were low; In addition, the *in vivo* half-life of these expressed rhFGF21s was short and the immunogenic activity was high, all which have restricted its clinical applications [5], [13]. In our previous study [13], fusion of SUMO (small ubiquitin-like modifier) to rhFGF21 has significantly enhanced the expression level of rhFGF21, but its *in vivo* biostability remains a problem.

Molecular modification of proteins with synthetic macromolecules such as poly(ethylene glycol) (PEG) has been extensively used to improve protein's biostabilities [14]–[16]. Reportedly PEGylated interleukin-2 significantly increased its potency against tumors *in vivo* [14]. PEGylated interferon-β-1b also became highly stabile, soluble, potent, and less immunogenic [15]. However, the modification of protein by PEG via non-selective groups did not result in homogeneous products, to avoid which, site-directed PEGylation to specific groups of the proteins has been developed so that the definite numbers of PEGs could be coupled selectively to proteins. N-terminal residues of the peptide have been proposed as useful selective targets for PEGylation with an advantage of producing homogeneous products without change of the peptide structure [17], [18]. For example, PEG aldehyde derivative has highly specific affinity to N-terminal residue of the peptide [16].

In the present study, therefore, we modified rhFGF21 by PEGylation at the N-terminal residue with 20 kDa mPEG-butyraldehyde (mPEG20K), and generated a high purity of homogenous product of PEGylated rhFGF21. The *in vitro* and *in vivo* biostabilities of PEGylated rhFGF21 were significantly improved compared to the native rhFGF21. More importantly the PEGylated rhFGF21 afforded a significantly long effect on lowing blood glucose and lipid levels in a type 2 diabetic rat model.

## Results

### PEGylation and Purification of rhFGF21

**Figure 1:**
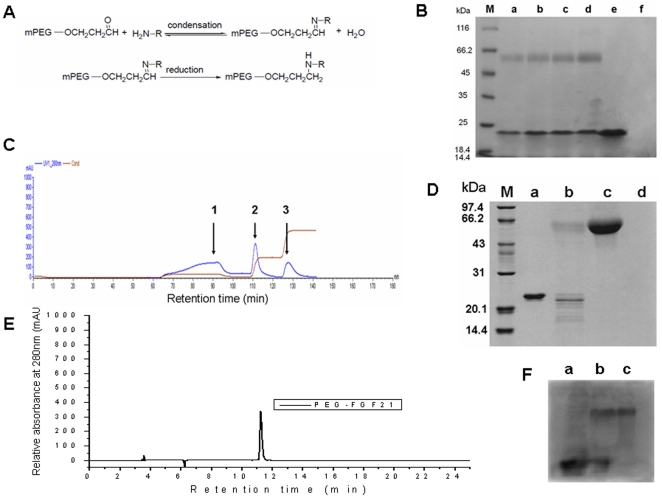
General information for PEGylated rhFGF21. Panel A: Schematic illustration of reaction of rhFGF21with PEG20 kDa-butyraldehyde. Panel B: SDS-PAGE analysis of PEGylation of FGF21 (lane M: molecular weight standards; lane a: reaction mixture of PEGylated rhFGF21 by incubating 1-fold molar ratio excess of 20 kDa mPEG-butyraldehyde to rhFGF21 in PBS, pH 6.0 at 4°C for 8 h; lane b: reaction mixture of PEGylated rhFGF21 by incubating 2-fold molar ratio excess of 20 kDa mPEG-butyraldehyde to rhFGF21 in PBS, pH 6.0 at 4°C for 8 h; lane c: reaction mixture of PEGylated rhFGF21 by incubating 3-fold molar ratio excess of 20 kDa mPEG-butyraldehyde to rhFGF21 in PBS, pH 6.0 at 4°C for 8 h; lane d: reaction mixture of PEGylated rhFGF21 by incubating 4-fold molar ratio excess of 20 kDa mPEG-butyraldehyde to rhFGF21 in PBS, pH 6.0 at 4°C for 8 h; lane e: native rhFGF21); lane e: native rhFGF21; lane f: mPEG-butyraldehyde. Panel C: Ion exchange chromatogram of reaction mixture [Peak1: follow-through fraction of reaction mixture of PEG regent and rhFGF21; Peak2: fraction eluted with elution buffer A (0.1 M Tris-HCl, 80 mM NaCl, pH 8.5); Peak 3: fraction eluted with elution buffer B (0.1 M Tris-HCl, 120 mM NaCl, pH 8.5)]. Panel D: SDS-PAGE analysis of PEGylated rhFGF21 purified using ion exchange chromatography (lane M: molecular weight standards; lane a: native rhFGF21; lane b: the fraction of Peak 3 from ion exchange chromatogram in panel C (including native rhFGF21 and PEGylated rhFGF21); lane c: the fraction of Peak 2 from ion exchange chromatogram in panel C (PEGylated rhFGF21); lane d: the fraction of Peak 1 from ion exchange chromatogram in panel C. Panel E: RP-HPLC analysis of PEGylated rhFGF21 purified using ion exchange chromatography. Panel F: Western blotting analysis of PEGylated rhFGF21 with specific antibody against human FGF21 (lane a: native rhFGF21; lane b: reaction mixture of PEGylated rhFGF21; lane c: PEGylated rhFGF21).

The site-specific PEGylation of rhFGF21 with mPEG-butyraldehyde is illustrated in **Figure 1A**. SDS-PAGE analysis (**Figure 1B**) showed that no signal was seen for PEG alone on SDS-PAGE gel (lane f), and the highest yield of PEGylation of rhFGF21 was obtained by incubating 4-fold molar ratios of 20 kDa mPEG-butyraldehyde with rhFGF21 in PBS, pH 6.0 at 4°C for 8 h (lane d) as compared to those at other ratios (lanes a–c). Based on the proportion calculation of the PEGylated rhFGF21 band at about 60 kDa to the native rhFGF21 band at around 22 kDa, the amount of the modified rhFGF21 was up to 47.1% in total polypeptide input. PEGylated rhFGF21 was purified by cation exchange chromatography **(** **Figure 1C** **)** and be identified on SDS-PAGE gel **(** **Figure 1D**). HPLC analysis suggested that the purity of PEGylated rhFGF21 with a retention time of 11.24 min exceeded 95% **(** **Figure 1E** **)**. Western blotting analysis with specific anti-human FGF21 antibody **(** **Figure 1F** **)** showed that there were two groups of proteins to specifically react with anti-human FGF21 antibody in the reaction mixture (lane b): one as the native rhFGF21 (same as lane a) and one as the PEGylated rhFGF21 (same as lane c).

**Figure 2:**
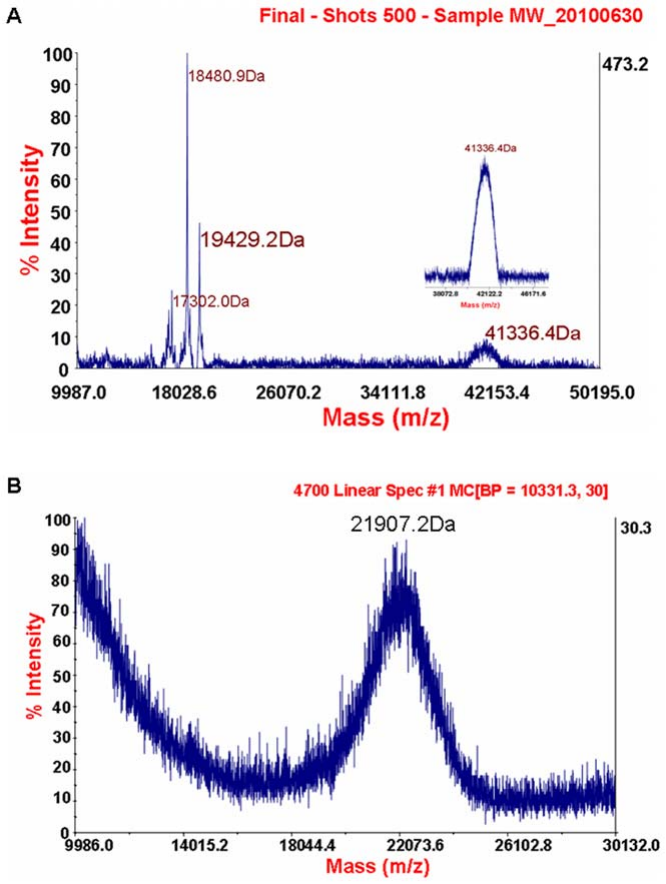
Identifying PEGlyated rhFGF21. MALDI-TOF mass spectroscopy of PEGylated rhFGF21 (A), showing the molecular mass of rhFGF21 as 19,429.2 Da and PEGylated as 41,336.4Da, and PEG (B), showing a broad peak of PEG molecular mass centering at 21,907.2 Da.

**Figure 3:**
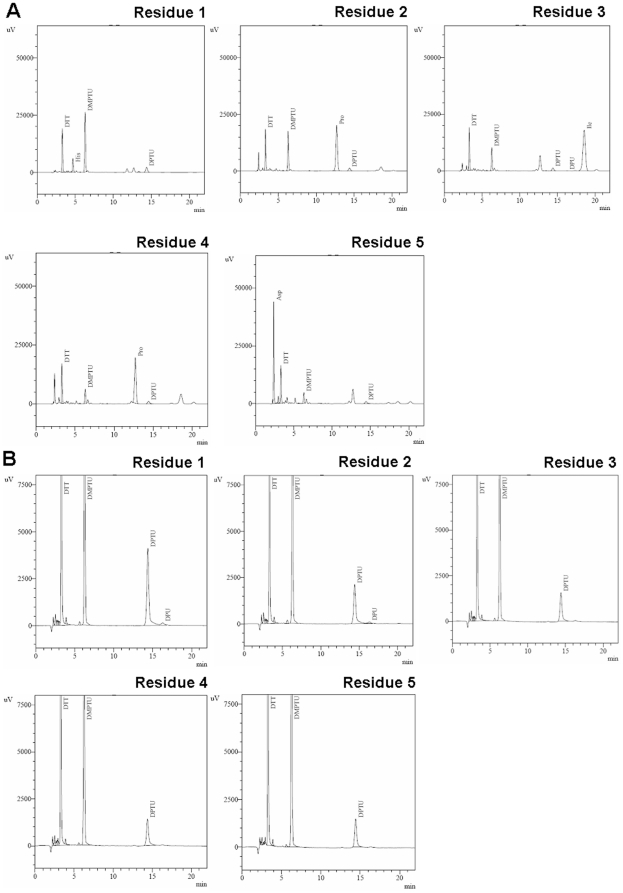
Comparison of N-terminal sequences. N-terminus Sequences of the native rhFGF21 (A) and PEGylated rhFGF21 (B)were compared by examining the status of Edman degradation.

The covalent conjugation of PEG-rhFGF21 was analyzed by MALDI-TOF mass spectroscopy. As shown in **Figure 2A**, a broad peak, centered at 41,336.4Da, was detected following rhFGF21 conjugation with PEG. The broad peak of PEGylated rhFGF21 was assumed due to PEG polydispersity as reported before [19]. To confirm this assumption, we showed that MALDI-TOF mass spectrum of PEG also had a broad peak, centered at 21,907.2 Da (**Figure 2B**). The evidence of PEG polydispersity also explains the occurrence of two peaks of 18,480.9 and 17,302.0 Da (**Figure 2A**) as other two PEGs except for the predominant component of 21,907.2 Da. It is known that the molecular weight of rhFGF21 is 19,424 Da [13]; therefore, the peak of 41,336.4 Da corresponds to a mono-PEGylated rhFGF21 (**Figure 2A**). Sequencing by Edman degradation revealed that the first five amino acids of native rhFGF21 N terminus were His-Pro-Ile-Pro-Asp (**Figure 3A**), but N-terminus of PEGylated rhFGF21 could not be degraded after four cycles (**Figure 3B**) since the covalently modified α-amine by PGE is resistant to 1-fluoro-2,4-dinitrobenzene cleavage [16]. The MS data and Edman degradation results strongly indicated the attachment of a PEG molecule to the N-terminus.

### Structural and generally biochemical evaluation of PEGylated FGF21

**Figure 4:**
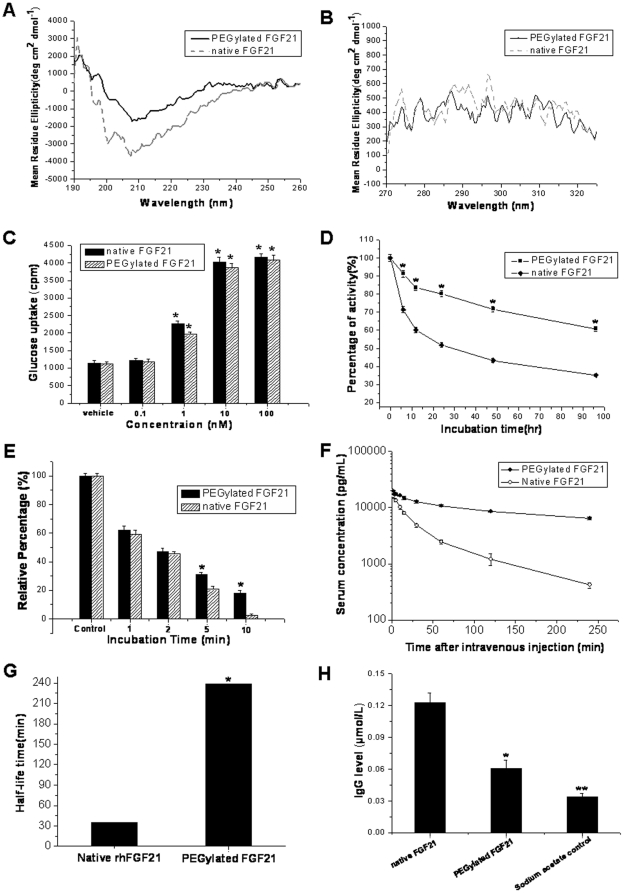
Structural and biochemical evaluation of PEGylated rhFGF21. Panel A: Far-UV CD spectra of native (dash line) and PEGylated rhFGF21 (black solid line). Panel B: Near-UV CD spectra of native (dash line) and PEGylated rhFGF21 (black solid line). The ellipticities are reported as mean residue ellipticitie (MRE) (deg cm^2^ dmol^−1^). Panel C: Cellular glucose uptake stimulated by native and PEGylated rhFGF21 on 3T3-L1, measured using Wallac 1450 MicroBeta counter (Perkin Elmer). Values (±SE) shown are the average of at least 3 independent measurements. *, P<0.001 vs vehicle control. Panel D: Thermal stability of native and PEGylated rhFGF21 incubated in mouse serum at 37 °C for indicated times, and then the serum-incubated rhFGF21 were added to 3T3-LI cells for which glucose uptake was measured to determine the functional integrity of each rhFGF21. Panel E: The relative resistance to trypsinization was examined, for which PEGylated and native rhFGF21 were incubated with trypsin at 2 mM for indicated times. Trypsin-treated rhFGF21s were examined for the protein integrity by SDS-PAGE. The bands representing PEGylated and native rhFGF21 were quantified by densitometry scanning. The band densities of non-trypsin-treated native and PEGylated rhFGF21 are considered as 100% (indicated by control), while the band densities of trypsin-treated PEGylated or native rhFGF21 are presented as relative percentage to the control. *, p<0.01 vs non-trypsin-treated native or PEGylated rhFGF21. Panel F: Pharmacokinetics study of PEGylated rhFGF21 in rat. Male Wistar adult rats were injected intravenously with 100 µg/kg native rhFGF21 (open circle), PEGylated rhFGF21 (close circle). Blood samples were collected at various time points. The amount of rhFGF21 was measured by ELISA. A standard curve was made for each rhFGF21. *n* = 5. Values are mean±SD. Panel G: Half-life time of rhFGF21 and PEGylated rhFGF21. Panel H. Immunogenicity of native and PEGylated rhFGF21 was examined by immunizing BALB/c mice with either PEGylated rhFGF21 or native rhFGF21 in 20 mM sodium acetate buffer by i.p. injection (2 µmol/mouse) twice (the 1^st^ day and the 14^th^ day) and then serum samples were collected on the 21^st^ day after the first immunization. Anti-rhFGF21 IgG levels were detected by indirect ELISA method using native FGF21 as the coating antigen. In addition, a group of mice given same volume of sodium acetate only for a same time schedule as rhFGF21 was included for sodium acetate control. *, p<0.01 and **, p<0.001vs native rhFGF21 group.

Circular dichroism (CD) spectroscopy analysis for the secondary and tertiary structures of PEGylated rhFGF21 shows that compared to the native rhFGF21, the far and near-UV CD spectra fingerprints of PEGylated rhFGF21 are comparable with that of native rhFGF21 **(** **Figure 4A and B** **)**, indicating that the interaction of PEG with protein does not compromise the protein structure. It should be noticed that the signal intensity across 190 nm to 240 nm is significantly greater for PEGylated rhFGF21 than that for native rhFGF21 (**Figure 4A**). In general, CD signal intensity reflects the concentration of protein samples so that if loading amounts of two protein samples have small difference, the intensities may differ slightly. Therefore, the fact that signal intensities across 190 nm to 240 nm are significantly different between native rhFGF21 and PEGylated rhFGF21 may be the slight difference for the loading amounts of these two proteins. We tested glucose uptake as the cellular function of the rhFGF21, which did not show a significant difference between the modified and native rhFGF21s (**Figure 4C**).

Next the thermal stability of both native and PEGylated rhFGF21 in mouse serum was examined by incubation at 37°C for different time-periods. The capacity of stimulating glucose uptake was significantly reduced for both native and PEGylated rhFGF21 after incubation with serum in a time-dependent manner. At 96 h after incubation, however, the native rhFGF21 retained only 37.8% of the original cellular bioactivity while PEGylated rhFGF21 retained 61.3% (**Figure 4D** **).** This assay indicates that PEGylation of rhFGF21 made it thermal tolerance compared to the native rhFGF21. The potent resistance of PEGylated FGF21 to proteolysis was also examined by incubation of two forms of rhFGF21 with trypsin at 2 mM for different times and then through electrophoresis on SDS-PAGE to see their integrities. At 10 min after incubation with trypsin, about 20% of PEGylated rhFGF21 retained intact while little of native rhFGF21 was left under same conditions (**Figure 4E**).

The *in vivo* half-life times of two forms of rhFGF21 were analyzed by intravenously injecting a single dose of 100 µg/kg of native or PEGylated rhFGF21 to male Wistar adult rats and then measuring the dynamic levels of two forms of rhFGF21 in the blood by ABC-ELISA method (**Figure 4F**). Based on the pharmacokinetic curves shown in Figure 4F, the eliminated plasma half-life (t1/2β) was calculated with Drug and Statistics Software (DAS, Version. 2.0; Mathematical Pharmacology Professional Committee of China) and the equation described in the *Materials and Methods*. The t1/2β of the unmodified rhFGF21 is about 35.1 min while the eliminated plasma t1/2β of PEGylated rhFGF21 is about 7 folds longer (**Figure 4G**).

To assess the immunogenicity of PEGylated FGF21, BALB/c mice were given by i.p. injection of PEGylated or native rhFGF21 (2 µmol/mouse) in 20 mM sodium acetate containing 1 ml FCA twice with an interval of 14 days. Serum samples were collected on the 21^st^ day after the first immunization, and the serum levels of anti-rhFGF21 IgG were detected by indirect ELISA with native rhFGF21 as the coating antigen. PEGylated rhFGF21 did not significantly induce anti-rhFGF21 antibody (IgG) in the plasma of the immunized mice compared to native rhFGF21 (**Figure 4H**).

### Anti-diabetic effects of PEGylated rhFGF21 in type 2 diabetic rats

**Figure 5:**
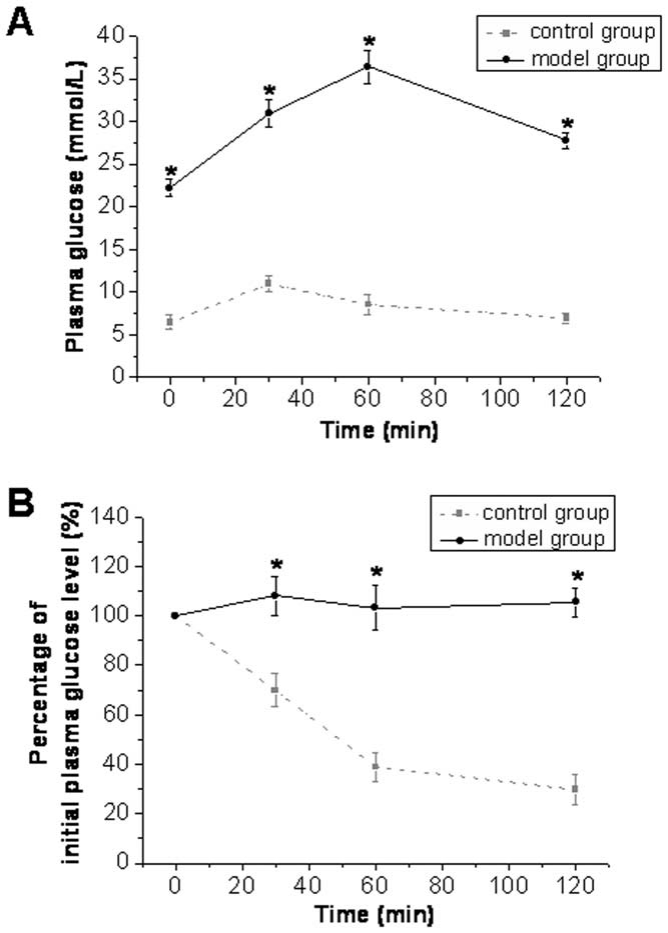
Examination for insulin resistance by IPGTT and IST. Type 2 diabetic rats were induced by 2-month HFD-feeding followed twice injection of low-dose of STZ at 35 mg/kg. At 8 weeks after STZ-induced hyperglycemia, IPGTT (A) and insulin sensitivity (B) were examined. For IPGTT, the rats with an overnight fast (12–16 h) were IP injected with 40% glucose (2 g/kg body weight) and blood samples were collected from the tail at 0, 30, 60, and 120 min for glucose measurement. For IST, insulin (0.75 IU/kg) was administered by IP injection and blood samples were collected at 0, 30, 60, and 120 minutes for the measurement of plasma glucose. The value is presented as a percentage of initial plasma glucose level.

The above biochemical analyses clearly indicate that PEGylated rhFGF21 was not different from the native rhFGF21 in terms of the *in vitro* bioactivity, but has a significantly higher biostability *in vitro* and *in vivo* than that of the native rhFGF21. These features prompt us to test whether PEGylated FGF21 can provide a better therapeutic effect on diabetes *in vivo* than the native rhFGF21. Type 2 diabetic rats were induced by 2-month high-fat-diet (HFD) feeding followed by two injections of low-dose STZ, by which the fasting blood glucose was significantly increased in diabetic group (15.63±0.69 mmol/L vs control [4.71±0.38 mmol/L], p<0.01). To ensure the induction of insulin resistance in these diabetic rats, intraperitoneal glucose tolerance test (IPGTT) and insulin sensitivity (IST) were performed at the 2 months after STZ-induced diabetes. As shown in **Figure 5A**, the glucose levels in diabetic and age-matched control rats increased to reach the highest level at 60 and 30 min, respectively, after injection of glucose (2 g/kg body weight), and then slowly decreased; however, diabetic rats were significantly hyperglycemic before and after glucose injection. IST showed that blood glucose levels declined rapidly in the control rats, but did not significantly change in the diabetic rats for the whole 120 min after insulin administration (**Figure 5B**). These results demonstrated that the rats at 2 months after STZ-induced diabetes presented a significant insulin resistance. All these data indicated that rats treated with HFD for 2 months followed by two injections of low-dose STZ developed a typical type 2 diabetes with insulin resistance and hyperglycemia (**Figure 5A,B**).

Diabetic rats with both hyperglycemia and insulin resistance were divided into two major groups: Diabetes and Diabetes/rhFGF21. In Diabetes/rhFGF21 groups, diabetic rats were treated by s.c injection of either native or PEGylated rhFGF21 at the dose levels of 1×10^−8^, 2×10^−8^ and 4×10^−8^ mol/kg daily for 7 days, based on a previous treatment regiment [5]. Body weight and food intake of diabetic rats before and after administration of the native or PEGylated FGF21 were monitored. As shown in Table 1, the body weight slowly increased in the control rats after treatment with rhFGF21 from 350.1 g before treatment to 380.2 g at the 7^th^ day after seven-day rhFGF21 treatment while the body weight in diabetic group was slightly decreased from 349.3 g to 326.5 g. Compared to control group, the body weights in the native rhFGF21-treated diabetic rats (TM1 group) were not changed before and after rhFGF21 treatment (Table 1); however, the body weights in PEGylated rhFGF21-treated diabetic rats (TM2) were slightly increased from 350.7 at before to 372.6 at the 7^th^ day after the seven-day rhFGF21 treatment, which is significantly different from native rhFGF21-treated group (Table 1, P<0.01). Table 1 also shows that diabetic rats increased their food intakes compared to control rats. Treatment of these diabetic rats with rhFGF21 reduced their food intakes. There was no difference between treatments with the native and PEGylated rhFGF21 during treatment, but the food intakes in diabetic rats treated with PEGylated rhFGF21 significantly less than that in the diabetic rats treated with native rhFGF21 at the 3^rd^ and 7^th^ day after seven-day treatment.

**Table 1:** Body weight and food intake of control group, DM group and TM group.

	Body weight (g)				Food intake (Kj/d)			
Time	CON	DM	TM_1_	TM_2_	CON	DM	TM_1_	TM_2_
0 week	197.2	200.4	199.3	201.2	34.5	35.6	35.1	35.7
Eight wks after diabetes	350.1	349.3	347.3	350.7	57.2	70.2	69.3	71.7
3^rd^ day during treatment	355.5	338.6	354.3	353.4	55.1	74.3	64.1	63.7
7^th^ day during treatment	365.1	334.2	361.3	363.2	58.8	79.2	61.3	60.9
3^rd^ day after 7-day treatment	369.7	326.5	355.6	367.6	64.3	84.7	67.2	62.2*
7^th^ day after 7-day treatment	380.2	326.5	347.6	372.6*	67.3	91.2	75.1	64.0*

CON: control group; DM: high-fat diet with STZ 35 mg/kg twice injection group; TM_1_: Native rhFGF21-treated diabetic rats; TM_2_: PEGylated rhFGF21-treated diabetic rats. The values shown are the average of the measurements of at least 5 animals in each group. *,p <0.05 vs corresponding TM_1_ (native rhFGF21) groups.

**Figure 6:**
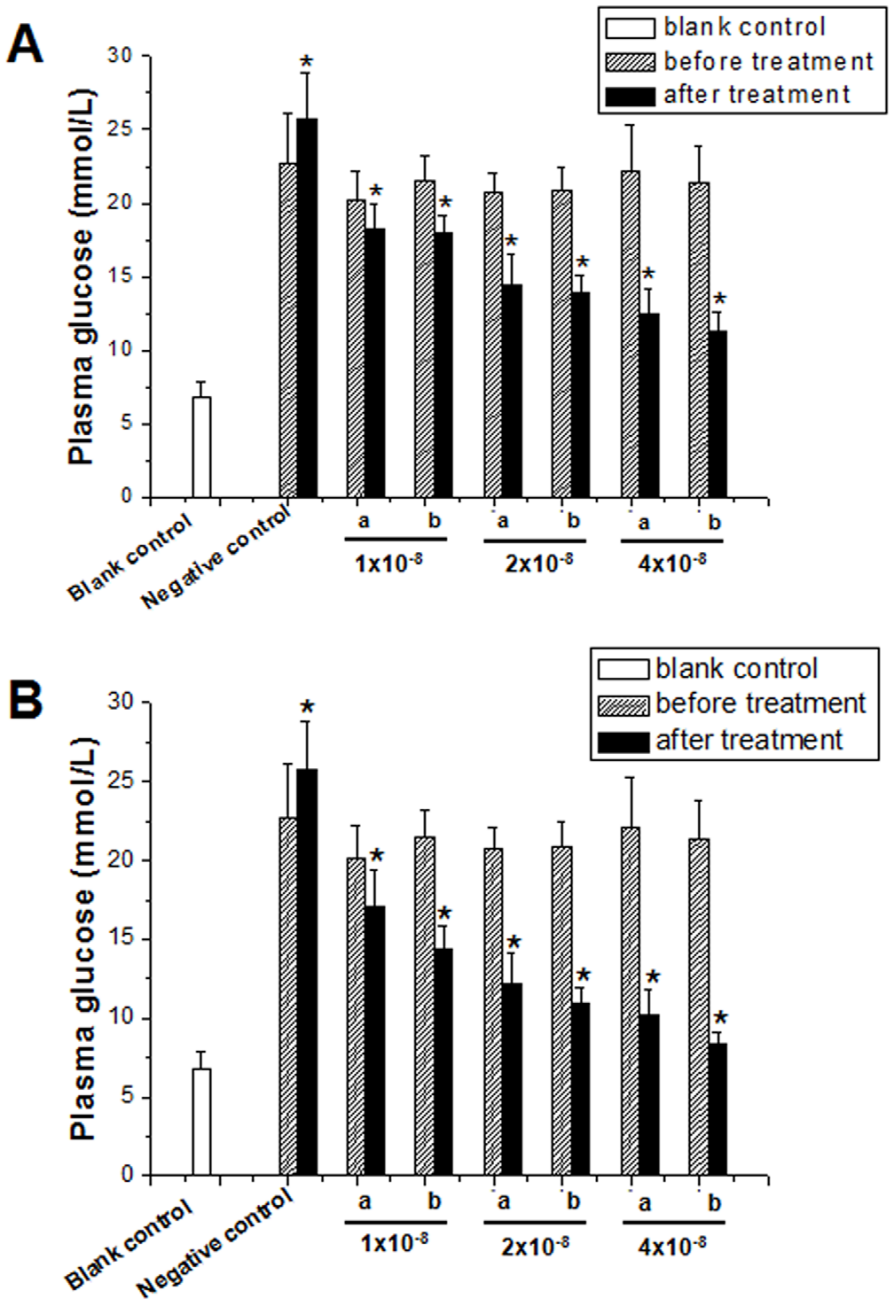
Anti-diabetic effects of PEGylated rhFGF21 in type 2 diabetic rats. After defined the induction of insulin resistance in these type 2 diabetic rats with IPGTT and IST assay as described in Fig 5, diabetic rats were treated with either native or PEGylated rhFGF21 at 1×10^−8^, 2×10^−8^, and 4×10^−8^ mol/kg daily for 2 weeks. On day 3 (A) and day 7 (B) after the first treatment of rhFGF21, plasma glucose levels were examined. Blank control: normal control rats; Negative control: 0.9% NaCl-treated diabetic group; a: native rhFGF21-treated diabetic rats; b: PEGylated FGF21-treated diabetic rats. *, P<0.01 vs corresponding pre-treatment groups.

**Figure 7:**
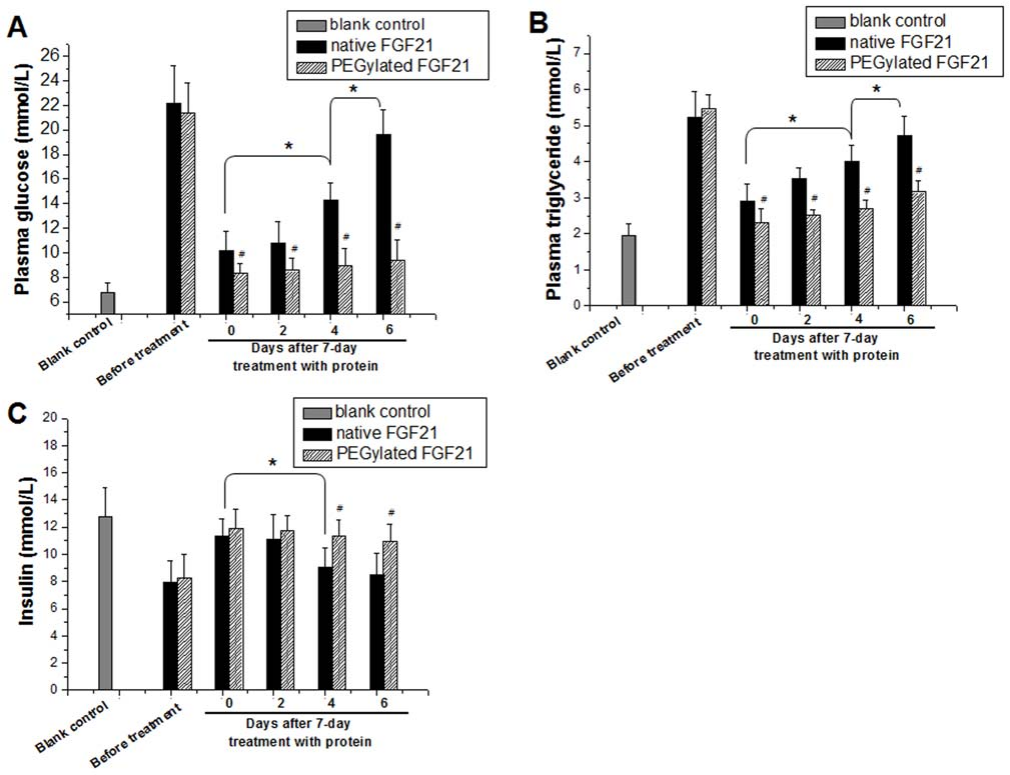
Long lasting anti-diabetic effects of PEGylated rhFGF21 in type 2 diabetic rats. Diabetic rat model and rhFGF21 treatment are same as Figure 5. However, hypoglycemic (A) and hypolipidemic effects (B) as well as effect on plasma insulin levels (C) of native and PEGylated rhFGF21 were examined at different times [0 (1 h), 2 days, 4 days and 6 days] after the last treatment of the 7-day treatment with native or PEGylated rhFGF21 at the 4×10^−8^ mol/kg. Blank control: normal control rats; #, p<0.01 vs corresponding native rhFGF21 group; *, p<0.05 between indicated groups.

Since systemic administration of FGF21 has been proved to significantly reduce plasma glucose and triglyceride in animal models of type 2 diabetes in previous studies [5], we measured the plasma glucose levels for all animals by tail blood at 1 h after the injection of rhFGF21 on day 3 (**Figure 6A**) and 7 (**Figure 6B**) during the treatment. Comparison of the glucose levels before and after treatment disclosed that both native and PEGylated rhFGF21 had significant hypoglycemic effects in a dose-dependent manner either on day 3 or day 7. Compared to native rhFGF21, however, PEGylated rhFGF21 afforded a slightly better hypoglycemic effect on day 7 during the treatment period (**Figure 6B**). The better hypoglycemic effect of PEGylated rhFGF21 than native FGF21 was considered due to the increased *in vivo* biostability of the modified rhFGF21.

To test the above assumption, we examined the plasma glucose (**Figure 7A**) and triglyceride (**Figure 7B**) levels at different times [immediately (0), 2 days, 4 days and 6 days] after seven-day treatment with rhFGF21 at the 4×10^−8^ mol/kg. Plasma glucose and triglyceride levels gradually returned to about pre-treatment levels in the diabetic rats treated with native rhFGF21, but remained at the low levels without significant difference from the level at day 0 in the rats treated with PEGylated rhFGF21. These results strongly support our hypothesis that increased *in vivo* biostability of PEGylated rhFGF21 provides significant and long-last hypoglycemic and hypolipidemic effects. Analysis of plasma insulin revealed that native rhFGF21 treatment significantly increased insulin levels, observed immediately after treatment, and gradually decreased to about pre-treatment levels at day 6 post-treatment, but PEGylated rhFGF21 not only increased insulin level immediately after treatment, but also remained it at relative high levels until day 6 (**Figure 7C**). This finding suggests that insulin level changes may be one reason in part for the difference between native and PEGylated FGF21 in terms of the long-lasting anti-metabolic effects.

## Discussion

Endocrine FGFs, including FGF19, FGF21, and FGF23, have emerged as novel endocrine regulators of multiple metabolic processes including energy and bile acid, homeostasis (FGF19) [20], glucose and lipid metabolism (FGF21) [11], phosphate, calcium and vitamin D homeostasis (FGF23) [21]. A feature of these ligands is the low receptor-binding and low heparin sulfate-binding affinity, as the molecular basis for the dependence of these ligands on Klotho proteins to signal in their target tissues [22]. In the other word, FGF21 requires β-Klotho as a cofactor to activate FGF receptor (FGFR) signaling [23], [24], [25], [26]. Micanovic *et al.* [27] and Yie *et al.* [28] further confirmed that the C-terminus of FGF21 is critical for FGF21 binding to β-Klotho and the N-terminus is critical for FGFR activation to initiate intracellular signaling. Therefore, our modification of N-terminal residue specifically with PEGylation should not affect its capacity to β-Klotho. N-terminal residue of the peptide has been proposed as a useful selective target for PEGylation with an advantage of producing homogeneous product without change of peptide structure [17], [18]. By CD spectroscopy analysis, we confirmed that there was no significant change for the secondary structure between PEGylated and native rhFGF21 (**Figure 4A,B**). Theoretically, this modification should not affect its intracellular action, which was confirmed in the present study by examining its effect on glucose intake in 3T3-L1 adipocytes (**Figure 4C**), consistent with other study that showed no impact on the protein function by the PEGylation [29].

In the present study, we also found that the molecular weight corresponding to the PEGylated FGF21 seemed relatively larger than the theoretical molecular size (Figure 1B,D). This phenomenon may be explained by the lower mobility of the PEGylated proteins during electrophoresis on SDS-PAGE as observed in a previous report [30].

PEGylation may affect physical, chemical and/or pharmacokinetic features of the proteins by steric hindrance and conformational change [31], [32]. In the present study, we demonstrate that PEGylated rhFGF21 becomes not only resistant to incubation with serum under body temperatures (**Figure 4D**) and to trypsinization (**Figure 4E**), but also weakly immunogenic (**Figure 4H**), compared to native rhFGF21. The specific binding of PEG to rhFGF21 may affect the heat- and trypsin-sensitive sites. Since trypsin as a typical protein proteolytic enzyme has been extensively used for investigation of the protein resistance to enzymatic digestion [16], [33]; therefore, we also selected it in the present study. Regarding the phenomenon that no difference for the resistance to trypsinization at the first 2 min between native and PEGylated rhFGF21, as shown in Figure 4E, we do not have exact explanation based on our knowledge, which will be further explored in the future studies.

Reduced immunogenicity of PEGylated FGF21 may be due to both the presence of PEG chains that envelops protein surface to restrict the accessibility of host IgG to the antigenic moiety of rhFGF21 to form specific antibody against rhFGF21 and the decrease in PEGylated rhFGF21transportability from blood to immune tissues such as spleen, bone marrow, and lymphoid tissue [34].

Kharitonenkov *et al.* [11] have demonstrated that *in vivo* half-life of FGF21 was 0.5 h if it was given by intravenous injection. Consistent with this finding, we showed that the half-life of rhFGF21 is about 0.5 h (35. 1 min., Figure 4G) when it was given by intravenous injection. However, the PEGylated rhFGF21 with the improved biostabilities described above has an *in vivo* half-life time about six-times longer (238.7 min, Figure 4G). The elongated half-life time of FGF21 by PEGylation may be explained by slower clearance from the circulation due to its larger size. In addition, conjugation of mPEG to FGF21 causes a size increase that may decrease filtration through the glomeruli of the kidney, whereas the unmodified protein may be filtered by the glomeruli [35].

Another important finding of the present study is the physiological confirmation that PEGylated rhFGF21 provided a more efficient effect, than native rhFGF21, on lowering blood glucose and triglyceride levels in the type 2 diabetic rat model (**Figure 7**). In a previous study [5] that is comparable with our study, *ob/ob* mice with both hyperglycemia and insulin resistance were given with FGF21 by s.c. once daily with 125 or 750 µg/kg/d for 7 days. Fed glucose levels were determined 1 h after administration FGF21 on day 3 and 7 during the treatment as we did here. Both doses of FGF21 significantly lowered blood glucose levels compared to vehicle treatment for 3 days. The effect was even more pronounced on day 7 during the treatment, making the fed glucose levels nearly normal levels in both dose groups [5]. All these features were confirmed in the present study with a type 2 diabetic rat model induced by HFD followed by low doses of STZ (**Figure 6**).

In the present study, the body weight slowly increased in control groups (from 355.5 g to 380.2 g), but slightly decreased in diabetic groups (from 349.3 g to 326.5 g) from eight weeks after diabetes to the 7^th^ day after seven-day rhFGF21 treatment (Table 1). Treatment with the native rhFGF21 only prevented the slightly decrease in body weight from the 8^th^ week after diabetes to the 7^th^ day after seven-day rhFGF21 treatment, while the treatment with PEGylated rhFGF21 not only prevented the decrease in body weight, but also increased body weight (from 350.7 g 372.6 g) during the same period (Table 1). In terms of food intake, mice from control and diabetes groups gradually increased from the 8^th^ week after diabetes to the 7^th^ day after seven-day rhFGF21 treatment, while mice from native rhFGF21 treated-group increased a small amount (from 69.3 to 75.1 Kj/d), but mice from PEGylated FGF21-treated decreased a small amount (from 71.7 to 64.0 Kj/d, Table 1). This is not consistent with the observed in previous studies showing that administration of FGF21 decreases body weight and body fat contents through increasing energy expenditure in obese mice [12], [36]. This discrepancy is probably due to diabetic models: we used HFD/STZ-induced diabetic mode that decrease body weight during experimental period while the two previous studies used HFD-induced obese and ob/ob mice, both which gained body weight during experimental period [12], [36].

An important finding in the early study is the potentially sustained effect of FGF21 on lowering blood glucose [5]. They found that fed glucose levels in FGF-treated animals were 25–35% lower than in the non-treated ob/ob mice at 24 h after last dose of the seven-day FGF-21 administration. This potentially sustained glucose-lowing effect of native rhFGF21 was confirmed and further extended by the present study. We found not only sustained glucose-lowing effect, but also sustained triglyceride-lowering effect at 24 h (day 2 in **Figure 7A,B**), and even at 72 h (day 4 in **Figure 7A,B**) after seven-day treatment with PEGylated rhFGF21 for the HFD/STZ-induced type 2 diabetic rats. More remarkably, both plasma glucose and triglyceride levels remained staying at very low levels without significant change even at the 6^th^ after the seven-day PEGylated rhFGF21 treatment (**Figure 7A,B**). Regarding the mechanism for the long-last lowing glucose effect, it remains unclear based on our current knowledge.

However, the slight increase in plasma insulin levels, as shown in **Figure 7C**, may be one of the mechanisms responsible for the long-last lowing glucose effect of PEGylated FGF21. Several previous studies have demonstrated the decrease in plasma insulin levels after acute or short-term administration of FGF21 [5], [12], [37]; however, constant administration of FGF21 could increased serum insulin level, along with a significant improvement of pancreatic β-cell function and survival [9]. The other studies also showed the protection of pancreatic β-cells by administration of FGF21 against various challenges [38]. In the present study, we demonstrated the increase in plasma insulin levels in the diabetic rats during and even at short time (3 days) after seven-day treatment with both forms of rhFGF21 (**Figure 7C**). This is the first study using diabetic model related to STZ that specifically damage pancreatic β-cells. Therefore, whether the increased insulin effect observed in the present study may be mediated by FGF21's improvement of pancreatic β-cell function and survival in response to the low-dose STZ-induced damage, as observed in other studies [9], [38], is warranty to be further investigated in the future studies.

In conclusion, to increase its *in vivo* life-time, stability, and therapeutic potency, we modified rhFGF21 for the first time by site-specific PEGylation with mPEG20kDa-butyraldehyde at N-terminal residue. The PEGylated rhFGF21 was purified to homogeneity by Q Sepharose anion-exchange chromatography. The mono-PEGylated rhFGF21 was found to retain the secondary structure, consistent with the native rhFGF21, and its biostabilities, including the resistance to physiological temperature and trypsinization, was significantly enhanced. PEGylated rhFGF21's immunogenicity *in vivo* was significantly decreased, and *in vivo* half-life time was significantly elongated. Compared to its native form, PEGylated rhFGF21 has similar bioactivity of stimulating glucose uptake in 3T3-L1 cells *in vitro*, but conferred a significant long-lasting effect on lowering blood glucose and triglyceride levels in type 2 diabetic animals. These results suggest that PEGylated rhFGF21 is a better and more effective anti-diabetic drug-candidate than the rhFGF21 currently available, which may be potentially applied in clinics for improving metabolic syndrome for type 2 diabetic patients.

## Materials and Methods

### Reagents

mPEG20 kDa-butyraldehyde was purchased from Sigma-Aldrich (St. Louis, MO, USA). Sepharose G50 column, Q Sepharose column, and AKTA purifier were purchased from GE Healthcare (Stockholm, Sweden). Proteins of rhFGF21 were produced by Engineering Research Center of Bioreactor and Pharmaceutical Development, Ministry of Education, Jilin Agricultural University (China), and anti-hFGF21 antibody was purchased from Santa Cruz Biotechnology Inc (Santa Cruz, CA, USA). The Bradford protein assay reagents used for quantitative protein analysis were purchased from Bio-Rad (Hercules, CA, USA). All chemicals were of analytical grade.

### Site-specific PEGylation of rhFGF21

The site-specific PEGylation of rhFGF21 with mPEG20K is illustrated in Figure 1A. Modification conditions were optimized following a factorial design. The factors, including molecular ratio of mPEG20K to the polypeptide, pH value, reaction temperature and reaction time, were optimized for the modification and summarized in Table 2. Reaction was terminated by adding 2% (w/v) glycine.

**Table 2:** Factors and levels of orthogonal tests.

	Molar ratio(FGF21:PEG)	pH value	Temperature (°C)	Reaction time (h)
1	1∶1	5.0	4	2
2	1∶2	6.0	25	4
3	1∶4	7.0	37	8

The reaction mixture was subjected to 12% SDS-PAGE. The gels stained with Coomassie Blue were scanned by optical density scanner (Beckman AppraiseT Densitometer, USA) to obtain modification rates.

### Purification of PEGylated rhFGF21

A Q Sepharose Fast Flow column was equilibrated with 15 column volumes (CVs) of binding buffer (0.1 M Tris-HCl, pH 8.5). The reaction complex was applied to the column. Sample was washed with 1 CV of binding buffer, and then eluted with elution buffer A (0.1 M Tris-HCl, 80 mM NaCl, pH 8.5) and elution buffer B (0.1 M Tris-HCl, 120 mM NaCl, pH 8.5) over 5 CVs. All elution fractions were collected and analyzed by SDS-PAGE.

The purity of PEGylated rhFGF21 was further examined by Reverse Phase High Performance Liquid Chromatography (RP-HPLC) analysis. The conditions of RP-HPLC were as following: Agilent 1100 RP-HPLC (Agilent Technologies, Palo Alto, CA) equipped with an automatic injector and an Eclipse XDB-C18 column. The mobile phase comprised two buffers: Buffer A (distilled H_2_O, 0.1% TFA, pH 2.0) and Buffer B (acetonitrile, 0.1% TFA). The RP column was first subjected to an isocratic 20% v/v acetonitrile-water gradient for 10 min, followed by a 20 to 80% (v/v) acetonitrile-water gradient over 60 min, at a total solvent flow-rate of 1 ml/min. Absorbance was measured at 280 nm using a UV detector.

### Analysis with mass spectroscopy

Mass spectra were acquired using an Applied Biosystems Voyager System DE PRO MALDI-TOF mass spectrometer (Carlsbad, CA, USA) with a nitrogen laser. The matrix was a saturated solution of R-cyano-4-hydroxycinnamic acid in a 50∶50 mixture of acetonitrile and water containing 0.1% trifluoroacetic acid. Purified PEGylated rhFGF21 and matrix were mixed at a ratio of 1∶1, and 1 µL was spotted onto a 100-well sample plate. All spectra were acquired in positive mode over the range 600-2500 Da under reflectron conditions (20 kV accelerating voltage, 350 ns extraction delay time) and 2-100 kDa under linear conditions (25 kV accelerating voltage, 750 ns extraction delay time). N-terminal amino acid sequence of PEGylated rhFGF21 was examined by Edman degradation method [39], and followed by MALDI-TOF mass spectroscopy as above.

### Cell culture and glucose uptake experiments

The 3T3-L1 preadipocytes (American Type Culture Collection, Rockville, MD, USA) were maintained in DMEM containing 10% fetal bovine serum (Invitrogen, Carlsbad, CA). Differentiation to adipocytes was induced by culturing the cells for 2 days in differentiation medium [DMEM/10% FBS/10 mM HEPES/MEM nonessential amino acids (NEAA)/penicillin/streptomycin (PC/SM)/2 µM insulin/1 µM dexamethasone/0.25 mM 3-isobutyl-1-methylxanthine (IBMX) (all from Sigma–Aldrich, St. Louis, MO, USA)] and then culturing in differentiation medium without dexamethasone and IBMX for another 2 days [24]. Thereafter, the medium was changed every 2 days with DMEM supplemented with 10% FBS/10 mM Hepes/NEAA/PC/SM. Accumulation of lipid droplets was observed in >95% of cells after 7 days, and the cells at day 7–10 were used for experiments.

For glucose uptake, cells cultured on multi-well plates were serum-starved overnight and then treated with different concentrations of either rhFGF21 or PEGylated rhFGF21 (0.1, 1, 10 and 100 nmol/L) for 24 h. The plates were washed twice with KRP buffer (15 mM HEPES, pH 7.4, 118 mM NaCl, 4.8 mM KCl, 1.2 mM MgSO_4_, 1.3 mM CaCl_2_, 1.2 mM KH_2_PO_4_, 0.1% BSA) and 100 µL of KRP buffer containing 2-deoxy-D-[^14^C]glucose (2-DOG)(0.1 µCi, 100 µM) was added to each well. Control wells contained 100 µL of KRP buffer with 2-DOG (0.1 µCi, 10 mM) to eliminate the non-specificity. The uptake reaction was carried out at 37°C for 1 h, terminated by addition of cytochalasin B (20 µM), and measured with Wallac 1450 MicroBeta counter (Perkin Elmer, Waltham, MA, USA).

### Circular dichroism spectroscopy

CD spectroscopy was used to determine the secondary and tertiary structure of native rhFGF21 and PEGylated rhFGF21. CD spectra were recorded on a Jasco J715 instrument using a cell of 0.02 cm path length. Spectral accumulation parameters included the scanning rate at 50 nm/min with a 2 nm bandwidth, over the wavelength range of 190–260 nm for far-UV CD measurements, and 270–320 nm for near-UV measurements. Each spectrum was obtained from an average of 10 scans. The CD data were presented in terms of the mean residue ellipticity (MRE) as a function of wavelength. The protein concentration of 0.5 mg/mL was used for each CD measurement. The CD spectra were corrected for buffer contributions.

### Effect of PEGylation on the thermal and structural stabilities of rhFGF21

To obtain the effects of PEGylation on the thermal stability of rhFGF21 at a physiological relevant temperature, PEGylated rhFGF21 and its non-modified control were incubated at a concentration of 0.01 mM at 37°C in mouse serum for different times as indicated. The samples containing 100 nmol/L of protein were then subjected to glucose uptake assay as described above. Similarly the effects of PEGylation on resistance of proteolysis of rhFGF21 were tested *in vitro* by incubating PEGylated and non-PEGylated rhFGF21 with trypsin (2 mM) in a molar ratio of 60∶1 (peptide:trypsin) at 37°C for different times indicated (Figure 4E). The samples were then subjected to SDS-PAGE gel electrophoresis to examine the protein integrity.

### Pharmacokinetic assay

Male Wistar adult rats (body weight 180–220 g) were used based on the procedures approved by the Animals Care and Use Committee from Jilin University [license No. SYXK(Ji)2002-0002]. The animal production licenses are No. SCXK 2003-0003. Rats were intravenously injected with 100 µg/kg body weight of native rhFGF21 or PEGylated rhFGF21 (five animals for each group). Blood samples were collected at different time points after protein administration via the tail vein. The blood samples were centrifuged and the serum was stored at −80°C. The concentrations of rhFGF21 were measured by an avidin biotin peroxidase complex enzyme-linked immunosorbent assay (ABC-ELISA) as described below. The pharmacokinetic parameters for native rhFGF21 and PEGylated rhFGF21 was calculated using Drug and Statistics Software (DAS, Version. 2.0; Mathematical Pharmacology Professional Committee of China). The elimination half-life (t1/2) was calculated using the formula t1/2 = 0.693/k_e_ (K_e_ stands for the elimination rate constant).

ABC-ELISA assay was performed by the following steps: the native rhFGF21or PEGylated rhFGF21 at different concentrations (0.4, 4, 40, 80 and 800 ng/mL) was added into 96-well plate to make a standard curve. For samples, serum sample (100 µl) collected from rat was coated onto 96-well plate in each well and incubated at 4 °C overnight. The plates both for standards and samples were washed with PBST three times and supplied with 100 µl of diluted rabbit anti-human rhFGF21 antibody (1∶1000) for each well. The plate was incubated at 37 °C for 1 h, then washed with PBST three times and supplied with 100 µl of secondary biotinylated antibody (1∶2000) in each well for 1 h incubation. The plate was washed with PBST three times and supplied with HRP-conjugated anti-biotin antibody (1∶2000), followed by washing and adding color-developing solution and reaction-stopping solution. The absorbance was measured by a spectrometer at 492 nm immediately. Standard curve was prepared with the concentrations of native rhFGF21or PEGylated rhFGF21 as x-axis and OD values as y-axis. The concentration of serum rhFGF21 or PEGylated rhFGF21 was calculated against the standard curve.

### Immunogenicity assay

Normal female BALB/c mice (18–21 g) were used based on the procedures approved by the Wenzhou Medical College Animals Care and Use Committee (license No. SYXK (Zhe) 2003-0003 and with the animal production license No. of SCXK 2003-1025. Mice were immunized with the native rhFGF21 or PEGylated rhFGF21 (n = 6) at dose of 2 µmol/mouse in 20 mM sodium acetate containing 1 ml Freund complete adjuvant (FCA) by intraperitoneal (i.p.) injection. Two weeks after the first immunization, mice were immunized once again with these two forms of rhFGF21. Serum samples were collected every 7 days after the first immunization. The specific IgG levels in serum were determined by ABC-ELISA, as described above.

### Type 2 diabetic model

Male Wistar adult rats (body weight 180–220 g) were used following the approved protocol and license No., as described above. Seventy-two rats were randomly divided into the high fat diet group (HFD, n = 64) and the common diet group (NC, n = 8). After 8-week HFD feeding, HFD group animals were given i.p. injections of 35 mg/kg STZ daily for two days, while the NC group was given an equivalent volume of citric acid buffer. One week later, blood was collected and the fasting blood glucose was assayed and the rats with the blood glucose levels >7.8 mmol/L were diagnosed as hyperglycemic.

For these diabetic and non-diabetic rats, i.p. glucose tolerance test (IPGTT) and insulin tolerance test (ITT) were further examined (see the next section). For IPGTT rats were fasted for 14 h and then IP injected with 40% glucose (2 g/kg body weight). Blood samples were collected from the tail at 0, 30, 60, and 120 min. for measurement of glucose. For ITT, insulin (0.75 IU/kg) was administered by I.P injection and blood samples were collected at 0, 30, 60, and 120 min. for the measurement of plasma glucose. The value is presented as a percentage of initial plasma glucose level.

### Anti-diabetic effects of PEGylated rhFGF21

Once hyperglycemia and insulin resistance were defined, 56 diabetic rats were divided into two major groups: control and rhFGF21-treated groups, based on a previous treatment regiment [5]. In rhFGF21 treated group, diabetic rats were injected s.c. once daily with either rhFGF21 or PEGylated rhFGF21 for 7 days at three dose levels: 1×10^−8^, 2×10^−8^ and 4×10^−8^ mol/kg (n = 8/group), and one group of diabetic rats treated with 0.9% NaCl group (non-treatment diabetes, n = 8). In addition, 8 normal rats were used as the non-diabetic control. On days 3 and 7 during the treatment, all animals were tail bled (by tail snip) at 1 h after administration the last injection of rhFGF21, and glucose level were determined using Precision G Blood Glucose Testing System (Abbott Laboratories). In addition, the long-last anti-diabetic effects of two forms of rhFGF21s were also compared by examining plasma glucose, triglyceride and insulin levels in the diabetic rats treated with rhFGF21 at 4×10^−8^ mol/kg at different times after rhFGF21 treatment. Plasma glucose and triglyceride levels were detected with Precision G Blood Glucose Testing System (Abbott Laboratories) and Hitachi 912 Clinical Chemistry analyzer (Roche Diagnostics Corp.), respectively. Plasma insulin levels were detected with murine Insulin ELISA kits (Crystal Chem Inc.).

### Protein estimation

Protein concentrations were measured with Bradford method [40] with BSA as the protein standard.

### Statistical analysis

Experimental data for glucose uptake experiments and *in vitro* study were obtained from at least three independent experiments with triple samples for each experimental condition. Data from animals study were obtained from five mice or six rats. Data was expressed as mean ± SD and subjected to statistical analysis by ANOVA and student *t*-test using statistical software NASDAQ: SPSS from SPSS Inc.
